# Supplementary material for: Development of an AI model for DILI-level prediction using liver organoid brightfield images
Source: Commun Biol. 2025 Jun 7;8:886. doi: 10.1038/s42003-025-08205-6 (PMC12145446; doi:10.1038/s42003-025-08205-6)
Supplement: Supplementary file 2 — Supplementary information [file 42003_2025_8205_MOESM2_ESM.pdf]

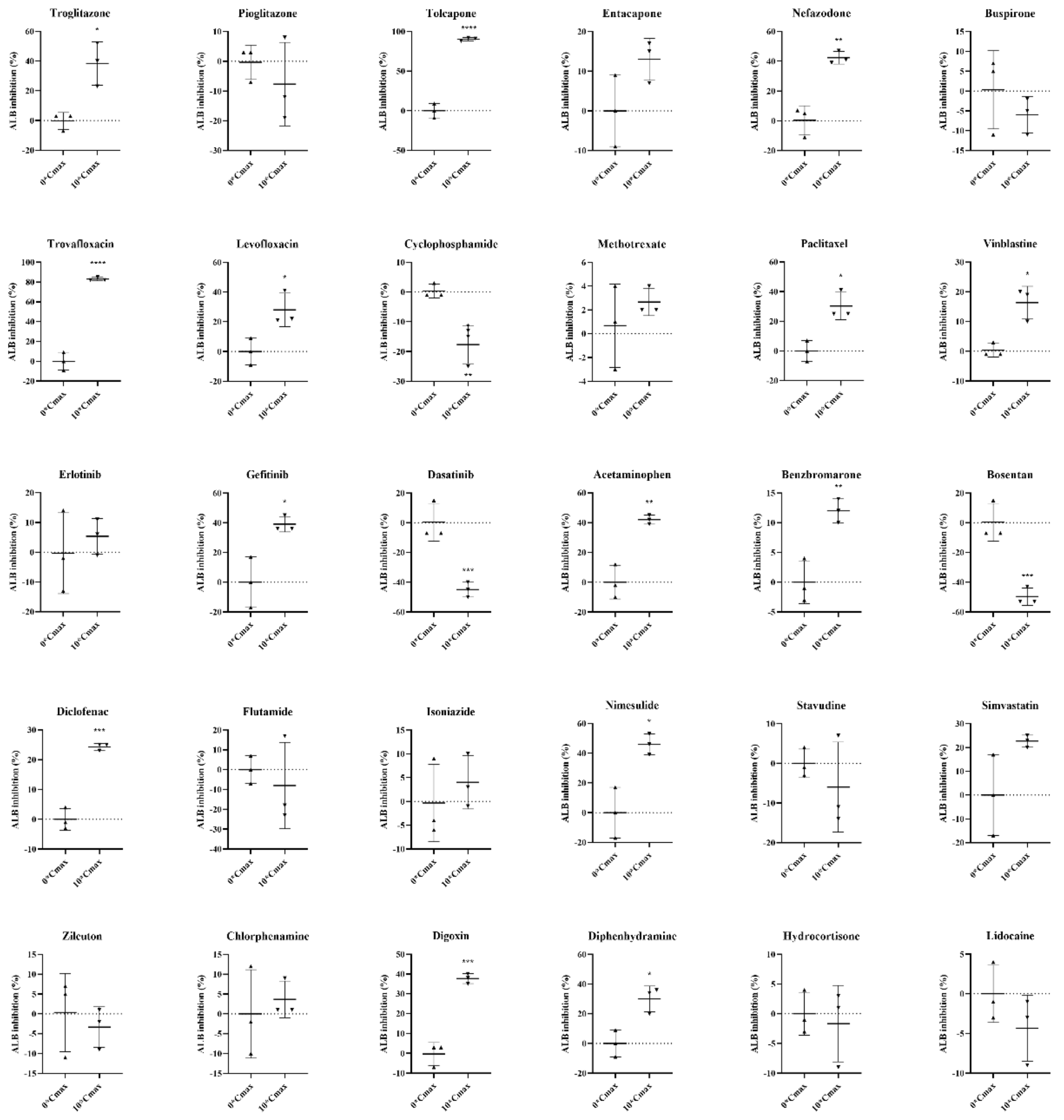

FigS1. ALB Inhibition (%) of Liver Organoids Treated with 0* and 10* Cmax of 30 Different Drugs of Hepatotoxicity or not. *, vs the Group of 0*Cmax, *, $P < 0.05$; **, $P < 0.01$; ***, $P < 0.001$; ****, $P < 0.0001$. n = 3.

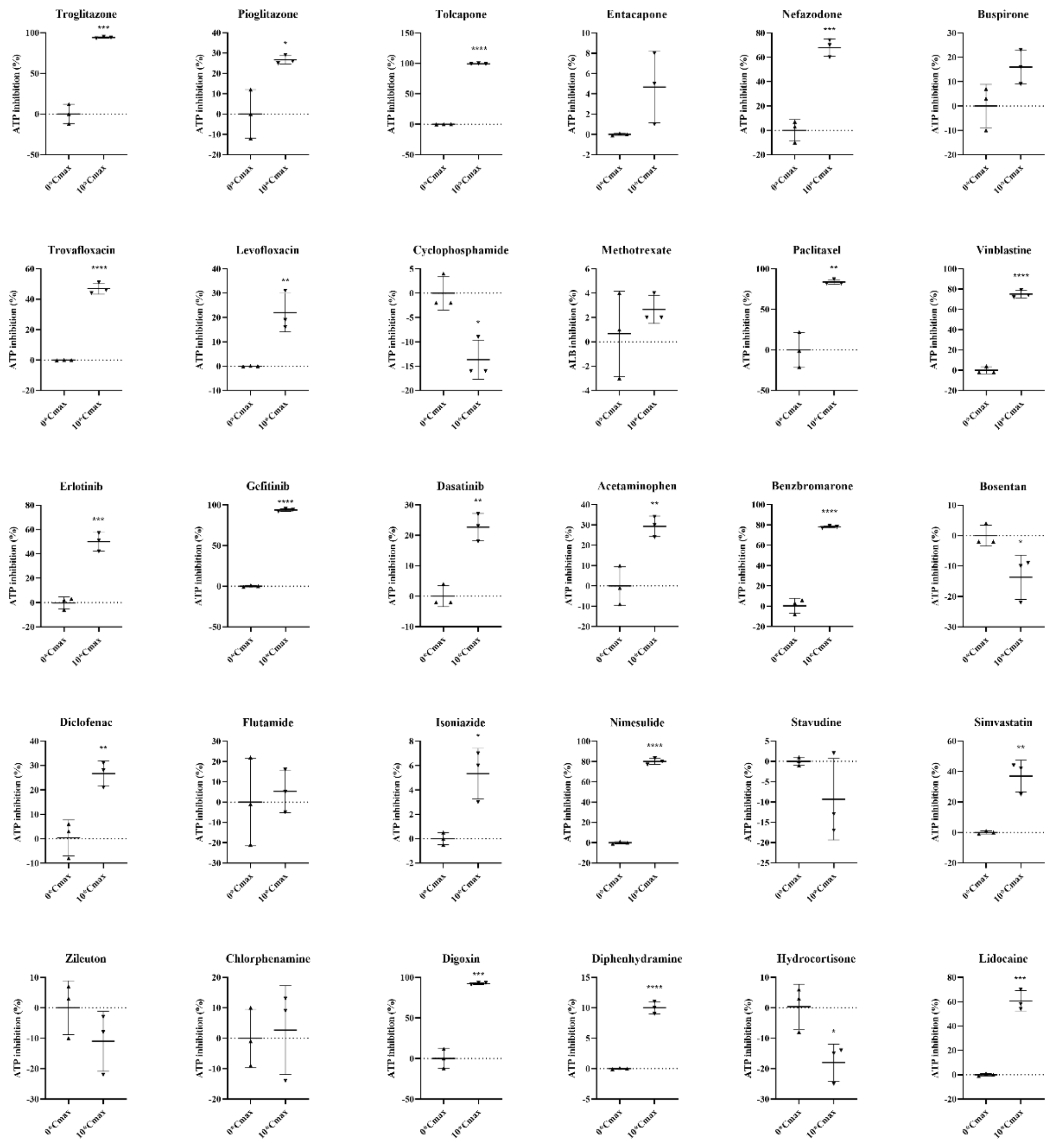

FigS2. ATP Inhibition (%) of Liver Organoids Treated with 0* and 10* Cmax of 30 Different Drugs of Hepatotoxicity or not. *, vs the Group of 0*Cmax, *, P < 0.05; **, P < 0.01; ***, P < 0.001; ****, P < 0.0001. n = 3.

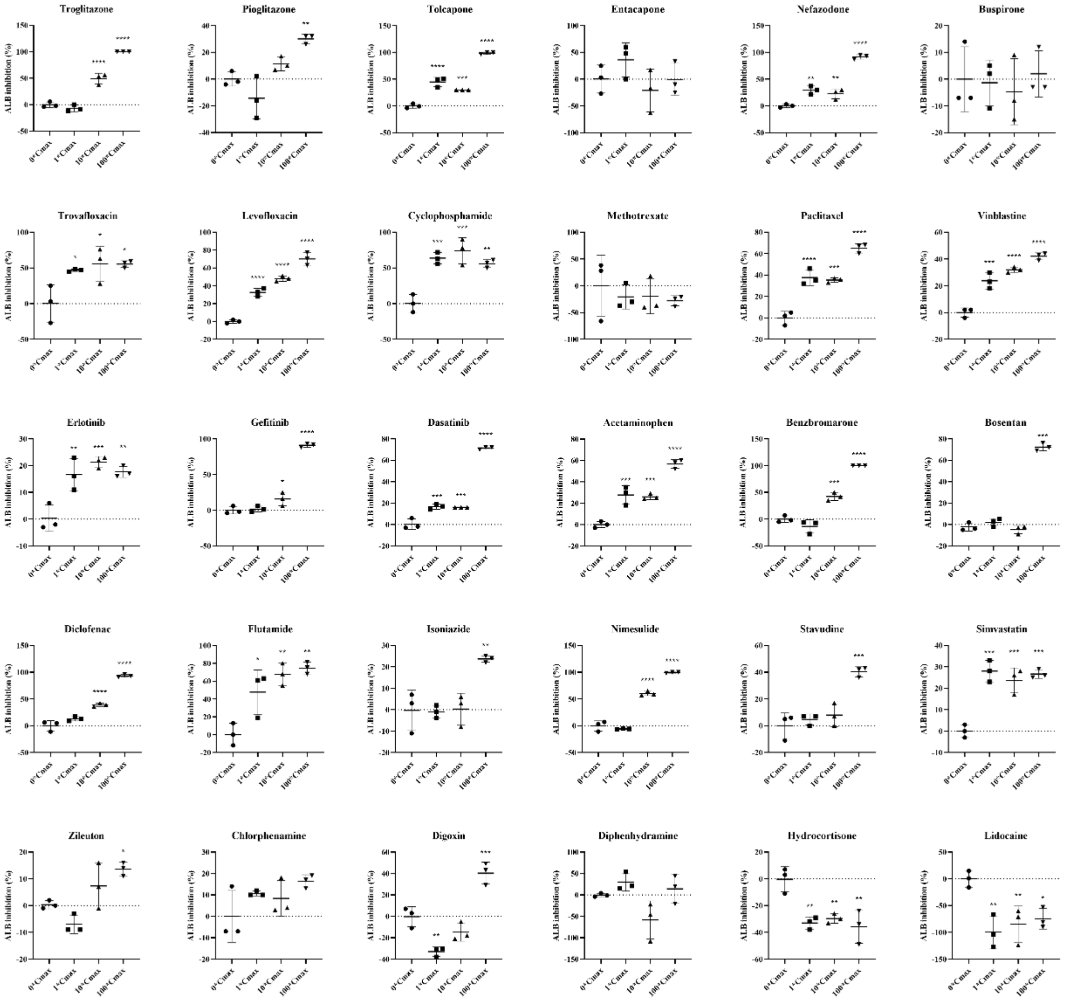

FigS3. ALB Inhibition (%) of HepG2 Spheroids Treated with 0*, 1*, 10*, 100*Cmax of 30 Different Drugs of Hepatotoxicity or not. *, vs the Group of 0*Cmax, *, $P < 0.05$; **, $P < 0.01$; ***, $P < 0.001$; ****, $P < 0.0001$. n = 3.

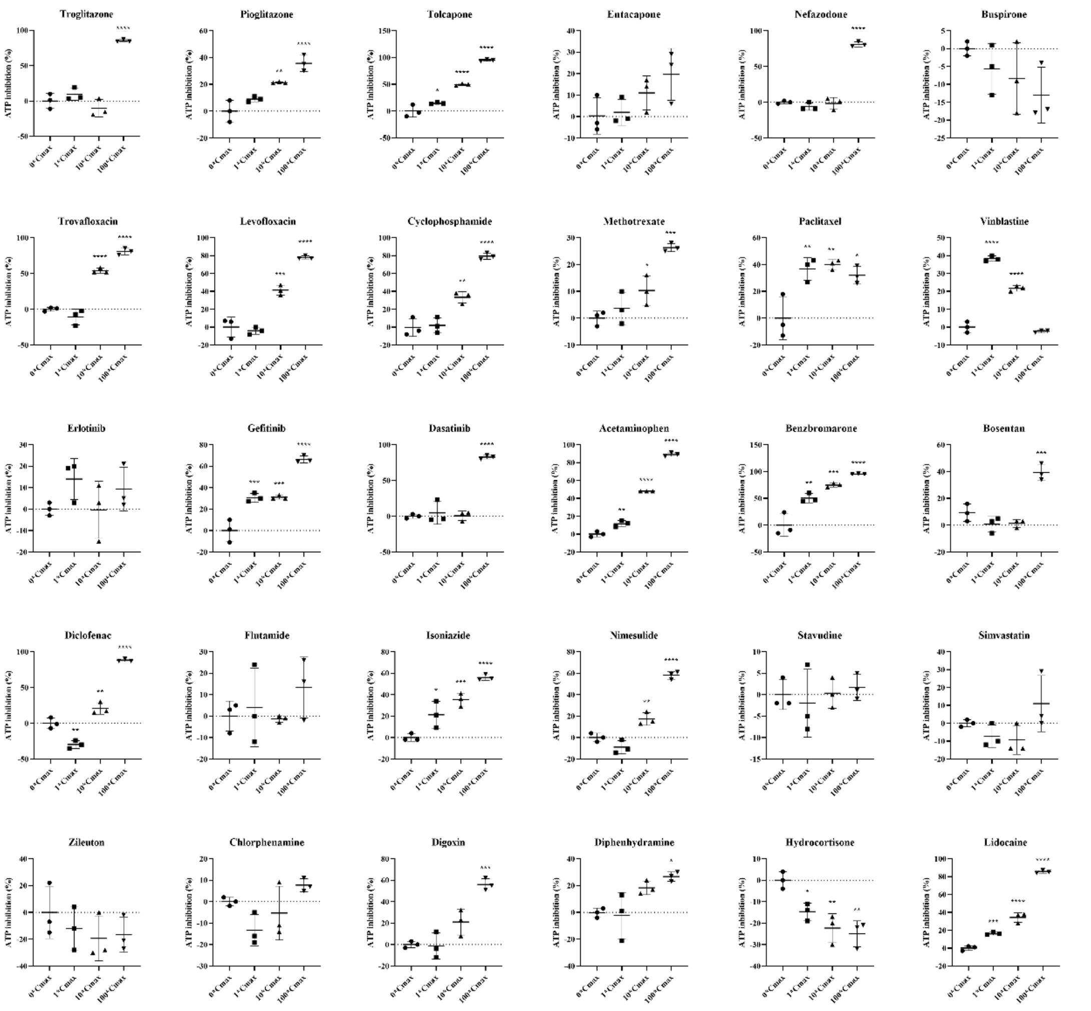

FigS4. ATP Inhibition (%) of HepG2 Spheroids Treated with 0*, 1*, 10*, 100*Cmax of 30 Different Drugs of Hepatotoxicity or not. *, vs the Group of 0*Cmax, *, $P < 0.05$; **, $P < 0.01$; ***, $P < 0.001$; ****, $P < 0.0001$. n = 3.

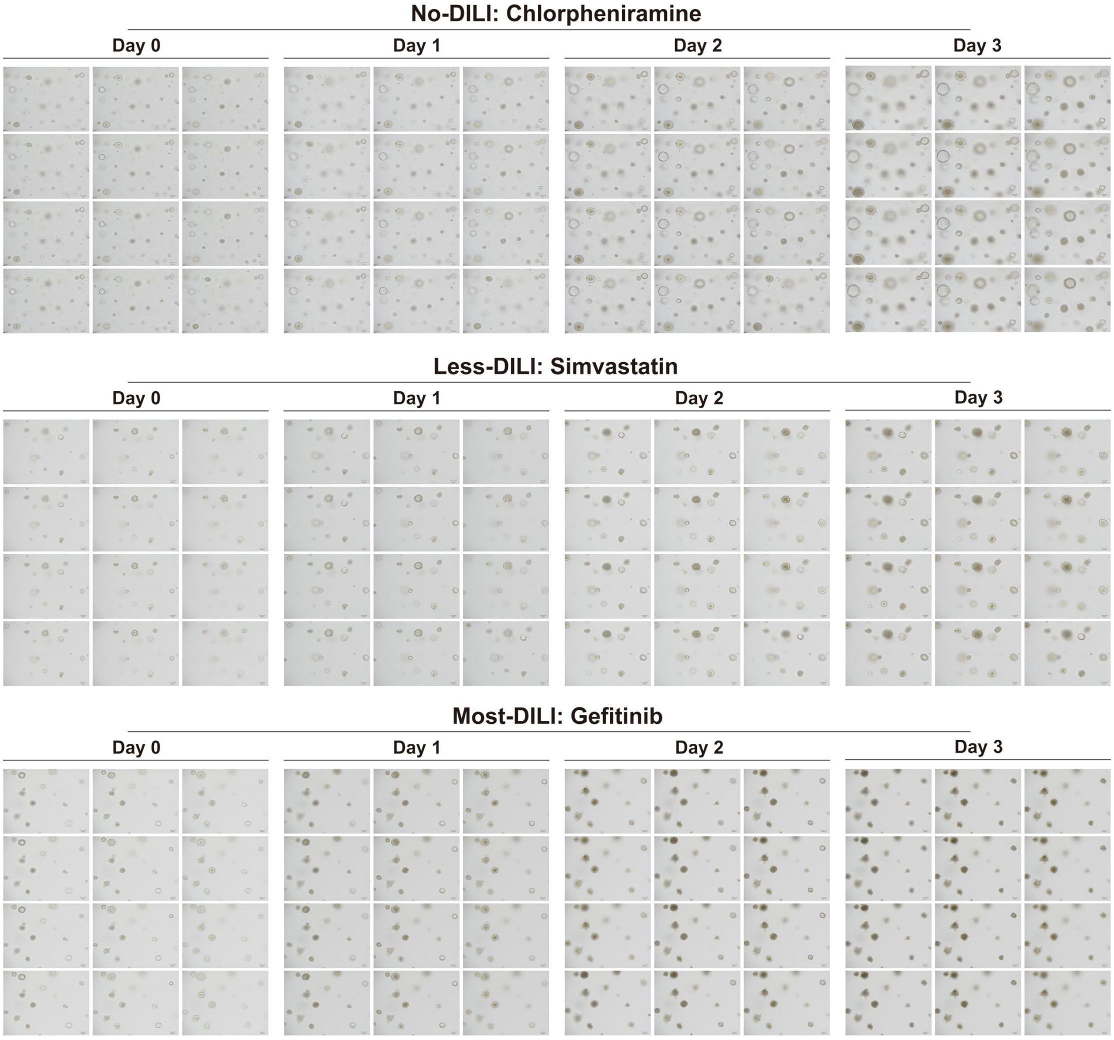

FigS5. Brightfield morphology changes (12 original images) of three compounds at different levels of liver injury in human liver organoids (Scale bar = 100 μm) from Day 0 to Day 3.

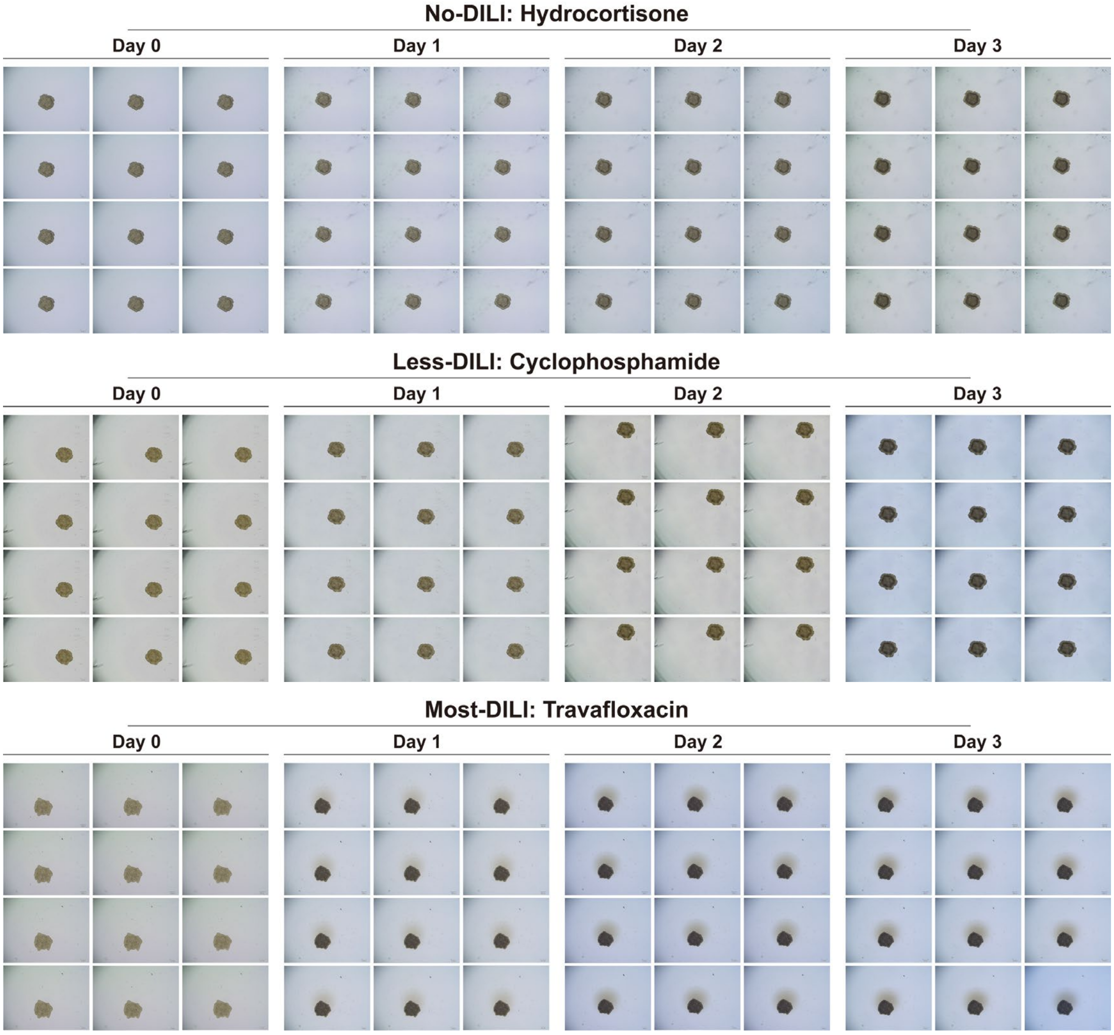

FigS6. Brightfield morphology changes (12 original images) of three compounds at different levels of liver injury in HepG2 spheroids (Scale bar = 250 µm) from Day 0 to Day 3.

Table S1. HepG2 spheroid based on ViLT model with a classification of drug concentrations.

	1*Cmax	10*Cmax	100*Cmax
5-fold cross-validation(1) Training set (best/last)	1/1	1/1	1/1
5-fold cross-validation(1) Test set (best/last)	0.72/0.68	0.75/0.71	0.70/0.60
5-fold cross-validation(2) Training set (best/last)	1/1	1/1	1/1
5-fold cross-validation(2) Test set (best/last)	0.72/0.56	0.75/0.54	0.75/0.60
5-fold cross-validation(3) Training set (best/last)	1/1	1/1	1/1
5-fold cross-validation(3) Test set (best/last)	0.80/0.68	0.83/0.75	0.65/0.65
5-fold cross-validation(4) Training set (best/last)	1/1	1/1	1/1
5-fold cross-validation(4) Test set (best/last)	0.72/0.6	0.75/0.46	0.65/0.45
5-fold cross-validation(5) Training set (best/last)	1/1	1/1	1/1
5-fold cross-validation(5) Test set (best/last)	0.73/0.59	0.64/0.59	0.69/0.50
Training set Ave (best/last)	1/1	1/1	1/1
Test set Ave (best/last)	0.73/0.62	**0.74**/0.61	0.69/0.56

Table S2. HepG2 spheroid based on iRENE model with a classification of drug concentrations.

	1*Cmax	10*Cmax	100*Cmax
5-fold cross-validation(1) Training set (best/last)	1/1	1/1	1/1
5-fold cross-validation(1) Test set (best/last)	0.68/0.48	0.83/0.63	0.65/0.65
5-fold cross-validation(2) Training set (best/last)	1/1	1/1	1/1
5-fold cross-validation(2) Test set (best/last)	0.92/0.76	0.75/0.63	0.70/0.70
5-fold cross-validation(3) Training set (best/last)	1/1	1/1	1/1
5-fold cross-validation(3) Test set (best/last)	0.76/0.68	0.83/0.83	0.75/0.75
5-fold cross-validation(4) Training set (best/last)	1/1	1/1	1/1
5-fold cross-validation(4) Test set (best/last)	0.76/0.64	0.67/0.58	0.90/0.80
5-fold cross-validation(5) Training set (best/last)	1/1	1/1	1/1
5-fold cross-validation(5) Test set (best/last)	0.73/0.55	0.82/0.73	0.75/0.63
Training set Ave (best/last)	1/1	1/1	1/1
Test set Ave (best/last)	0.77/0.62	**0.78**/0.68	0.75/0.71

Table S3. Cmax of different drugs (n = 30).

No	Compounds	Cmax	No	Compounds	Cmax
1	Troglitazone	2.82 μg/ml	16	Acetaminophen	21 μg/ml
2	Pioglitazone	1.05 μg/ml	17	Benzbromarone	1.84 μg/ml
3	Tolcapone	13 μg/ml	18	Bosentan	2.286 μg/ml
4	Entacapone	1 μg/ml	19	Diclofenac	2.367 μg/ml
5	Nefazodone	0.4349 μg/ml	20	Flutamine	0.1 μg/ml
6	Buspirone	0.00192 μg/ml	21	Isoniazid	10.5 μg/ml
7	Trovafloxacin	2.09 μg/ml	22	Nimesulide	6.5 μg/ml
8	Levofloxacin	5.7 μg/ml	23	Stavudine	0.7764 μg/ml
9	Cyclophosphamide	69 μg/ml	24	Simvastatin	0.01 μg/ml
10	Methotrexate	0.351 μg/ml	25	Zileuton	3.1 μg/ml
11	Paclitaxel	0.6 μg/ml	26	Chlorpheniramine	0.14 μg/ml
12	Erlotinib	1.2 μg/ml	27	Digoxin	0.00221 μg/ml
13	Gefitinib	0.2 μg/ml	28	Diphenhydramine	0.0876 μg/ml
14	Dasatinib	0.129 μg/ml	29	Hydrocortisone	0.305 μg/ml
15	Vinblastine	0.028 μg/ml	30	Lidocaine	8.5 μg/ml

Table S4. Primers Used in This Study

Gene	Forward	Reward
GAPDH	ACCCACTCCTCCACCTTTGA	CTGTTGCTGTAGCCAAATTCGT
CYP3A4	CATTCCTCATCCCAATTCTTGAAGT	CCACTCGGTGCTTTTGTGTATCT
CYP1A2	AGGTCAACCATGACCCAGAG	AGGGCTTGTTAATGGCAGTG
CYP2D6	TGCCGCCTTCGCCAACCACT	GGCTGGGTCCCAGGTCATAC
CYP2E1	CTGCAACGTCATAGCCGACA	TCCATTTCCACGAGCAGGCA

Table S5. Statistical parameters for ALB and ATP inhibition induced by drugs in organoids

Drugs	Indicators	*P* value	t value	Cohen's *d*	95% CI
Chlorpheniramine	ALB inhibition	0.6262	0.5258	0.43	-15.66, 22.99
	ATP inhibition	0.8040	0.2652	0.22	-25.25, 30.58
Simvastatin	ALB inhibition	0.0844	2.285	1.87	-4.881, 50.21
	ATP inhibition	0.0036	6.110	4.99	20.19, 53.81
Gefitinib	ALB inhibition	0.0191	3.800	3.10	10.50, 67.50
	ATP inhibition	<0.0001	88.86	72.57	90.74, 96.59

Note: $\text{Cohen's d} = t * \sqrt{\frac{2}{n}}$ in this study.

Table S6. Statistical parameters for ALB and ATP inhibition induced by drugs in spheroids

Drugs	Indicators	*P* value	t value	Cohen's *d*	95% CI
Hydrocortisone	ALB inhibition	0.0073	5.039	3.56	-45.50, -13.17
	ATP inhibition	0.0080	4.900	3.46	-34.99, -9.678
Cyclophosphamide	ALB inhibition	0.0045	5.750	4.07	38.10, 109.2
	ATP inhibition	0.0080	4.899	3.46	14.59, 52.75
Travafloxacin	ALB inhibition	0.0564	2.661	1.88	-2.411, 113.1
	ATP inhibition	<0.0001	21.46	15.17	47.01, 60.99

Note: $\text{Cohen's d} = t * \sqrt{\frac{2}{n}}$ in this study.

Table S7. Statistical parameters for CYPs in organoids and spheroids

CYPs	*P* value	t value	Cohen's *d*	95% CI
CYP3A4	0.0175	3.900	3.18	0.2319, 1.377
CYP1A2	0.0022	7.022	5.73	53.23, 122.9
CYP2D6	0.0021	7.108	5.80	2.419, 5.520
CYP2E1	0.0010	8.505	6.94	6.623, 13.04
CYP2C9	<0.0001	20.51	16.74	6.227, 8.177
CYP2C19	0.0031	6.374	5.20	4.562, 11.60

Note: $\text{Cohen's d} = t * \sqrt{\frac{2}{n}}$ in this study.
