# Supplementary material for: Development of an AI model for DILI-level prediction using liver organoid brightfield images
Source: Commun Biol. 2025 Jun 7;8:886. doi: 10.1038/s42003-025-08205-6 (PMC12145446; doi:10.1038/s42003-025-08205-6)
Supplement: Supplementary file 3 — Description of Additional Supplementary Files [file 42003_2025_8205_MOESM3_ESM.pdf]

Description of Additional Supplementary Files

File name: Supplementary Data 1

Description: The source data of Figure2 (b,d,f) in the paper.

File name: Supplementary Data 2

Description: The source data of Figure3 (b,d,f) in the paper.

File name: Supplementary Data 3

Description: The source data of Figure5 (b) in the paper.

File name: Supplementary Data 4

Description: CSV index file for the organoid image and HepG2 spheroid datasets. Each row corresponds to a single TIFF image and includes metadata such as sample ID, image filename, zaxis index, timepoint index, and DILI classification label.
